# A semi-supervised machine learning framework for microRNA classification

**DOI:** 10.1186/s40246-019-0221-7

**Published:** 2019-10-22

**Authors:** Mohsen Sheikh Hassani, James R. Green

**Affiliations:** Department of Systems and Computer Engineering, Carleton University, Ottawa, Ontario Canada

**Keywords:** Machine learning, Semi-supervised learning, Active learning, Co-training, miRNA prediction, Next-generation sequencing

## Abstract

**Background:**

MicroRNAs (miRNAs) are a family of short, non-coding RNAs that have been linked to critical cellular activities, most notably regulation of gene expression. The identification of miRNA is a cross-disciplinary approach that requires both computational identification methods and wet-lab validation experiments, making it a resource-intensive procedure. While numerous machine learning methods have been developed to increase classification accuracy and thus reduce validation costs, most methods use supervised learning and thus require large labeled training data sets, often not feasible for less-sequenced species. On the other hand, there is now an abundance of unlabeled RNA sequence data due to the emergence of high-throughput wet-lab experimental procedures, such as next-generation sequencing.

**Results:**

This paper explores the application of semi-supervised machine learning for miRNA classification in order to maximize the utility of both labeled and unlabeled data. We here present the novel combination of two semi-supervised approaches: active learning and multi-view co-training. Results across six diverse species show that this multi-stage semi-supervised approach is able to improve classification performance using very small numbers of labeled instances, effectively leveraging the available unlabeled data.

**Conclusions:**

The proposed semi-supervised miRNA classification pipeline holds the potential to identify novel miRNA with high recall and precision while requiring very small numbers of previously known miRNA. Such a method could be highly beneficial when studying miRNA in newly sequenced genomes of niche species with few known examples of miRNA.

## Background

MicroRNAs (miRNAs) are short (~ 18–25 nt), non-coding RNA (ribonucleic acid) sequences involved in cell regulation at both the post-transcriptional and translational levels. Regulation is achieved through inhibition of translation at the ribosome or targeting messenger RNA (mRNA) for degradation prior to translation. Studies have suggested that the majority of mRNA may be targeted by one or more miRNA [1], thereby implicating miRNA in cell cycle control [2], biological development [3, 4]^,^ differentiation [5], cancer biology [6–9] and other disease pathogenesis [10], stress response [11–13], and adaptation to environmental stresses [14, 15].

Clearly, the ability to identify miRNA within genomes is an important first step in understanding their function. *Computational* approaches to identifying miRNA are complementary to costly and resource-intensive wet-lab validation experiments such as northern blotting [16], RT-PCR [9] and microarrays [17].

A wide range of computational methods have been developed for the identification of miRNA directly from genomic sequence (i.e., de novo methods) or from next-generation sequencing data (i.e., NGS methods) [18, 19]. These techniques search the input data for pre-miRNA sequences forming miRNA-like hairpins and classify them based on computed sequence- or expression-based features. This task is made difficult by the high prevalence of pseudo-miRNA sequences within the genome that appear to fold into miRNA-like structures but do not lead to actual miRNA. This leads to significant class imbalance, where the number of true positives sequences is dwarfed by the number of negative (pseudo-miRNA) sequences. This is particularly true for de novo prediction methods, since they must consider all candidate pre-miRNA sequences and are not restricted to considering only those sub-sequences that are expressed in the cell, as is the case with NGS-based methods. However, de novo methods are more widely applicable since they do not require NGS transcriptomic data, only genomic sequence data.

Existing methods of miRNA identification rely on supervised machine learning (ML). In this paradigm, decision logic is learned directly from labeled training examples of both known miRNA and pseudo-miRNA. Effective classifiers require large quantities of labeled training data. However, for many species, there is a paucity of known miRNA, effectively limiting the accuracy of any supervised learning approach. For example, miRBase [20] contains experimentally validated miRNA sequences for less than 300 species. Furthermore, for approximately one third of such species, only 15 or fewer known miRNA sequences are available. Taken together, we conclude that there are insufficient training exemplars available for most species from which to train a miRNA classifier. While it is possible to train a miRNA classifier using data from a species that differs from the target species, we have previously shown that classification accuracy is reduced as the evolutionary distance between training and testing species increases [21].

Semi-supervised ML presents an opportunity to create more effective miRNA classifiers, in the face of limited labeled training data. Emerging high-throughput techniques, such as NGS, are able to produce vast quantities of data describing expressed RNA sequences. The difficulty in using these data to develop miRNA classifiers lies in the fact that they are unlabeled: we do not know if these expressed sequences represent true miRNA or if they come from other sources, such as mRNA degradation or processing. Semi-supervised ML is able to learn from both the small amount of available labeled training data and also from the much larger volume of unlabeled data. This study examines two such approaches: multi-view co-training and active learning.

The problem of miRNA prediction can be examined from two separate views, sequence-based or expression-based, resulting in two independent feature sets describing the same classification problem. Recent methods, such as miPIE [22] and mirnovo [23], have examined the use of integrated feature sets, that include both expression- and sequence-based features, and achieved substantial improvements in accuracy. However, the availability of two independent views of the problem enables the application of multi-view co-training (MVCT) approaches to semi-supervised ML [24]. In this approach, the available training data are used to create miRNA classifiers for each view separately. The classifiers are then applied to all available unlabeled data, and the highest confidence predictions are added to the training set of the alternate view. In this way, each view strengthens the classifier of the other view. This has been shown to be an effective way to avoid simply reinforcing the bias of a single classifier. Applications of MVCT within bioinformatics have been focused on the prediction of protein function [25], prediction of breast cancer survivability [26], detection of mis-localized proteins in human cancers [27], gene expression classification [28], cancer sample classification [29], and phenotype prediction [30]. We have recently investigated the use of MVCT for increasing the accuracy of miRNA classifiers [31]. In that study, classifiers were trained for each view independently. A consensus prediction is then achieved by confidence-weighted voting among the two views. In the present paper, we instead investigate the use of MVCT for augmenting the starting labeled training set for a second stage of semi-supervised learning using an integrated feature set.

While MVCT seeks to expand the available training data without any costly wet-lab validation, active learning seeks to identify those unlabeled samples that would be most beneficial to label, assuming that a limited budget is available for wet-lab validation experiments. Active learning is an iterative approach that begins by training a classifier using all available training data. The classifier is then applied to all unlabeled data and those points falling closest to the decision boundary are identified as candidates for subsequent experimental validation. By focusing on the points for which the classifier is most uncertain of their true class, maximal information can be gleaned for the classifier while minimizing wet-lab validation experiments. We have previously demonstrated the potential for active learning in miRNA classification [32]. In other areas of bioinformatics, active learning has been applied to drug discovery [33, 34], gene expression profiling of cancer biopsies [35] and histopathological images [36, 37], protein-protein interaction prediction [38, 39], and the identification of novel substrates for enzymes [40].

While both of these methods of semi-supervised ML have been shown to be effective in isolation, to our knowledge, they have not been explored in combination. The fact that MVCT focuses on adding unlabeled points to the training set for which the classifier is most confident, while active learning focuses on those unlabeled points for which the classifier is least confident, we hypothesize that these two methods are complementary. We hereby propose a novel semi-supervised approach for the classification of miRNA where a combination of active learning and multi-view co-training is used for increased classification performance.

We here conduct repeated cross-validation experiments to demonstrate that our proposed dual-stage semi-supervised approach reduces the number of labeled instances required in the training process thereby minimizing the overall cost of developing a miRNA predictor. Features are extracted from six diverse species to train and test predictors. The learning process consists of two stages, with an initial MVCT step followed by active learning. The individual contributions of each stage are quantified for each species and the combined pipeline is shown to be more effective than either MVCT or active learning applied separately. Final classification performance of the integrated semi-supervised pipeline, when constrained to using only 32 labeled training exemplars, is shown to surpass that of a state-of-the-art classifier trained with an unconstrained dataset.

This study represents the first published combination of MVCT and active learning into an integrated semi-supervised ML framework. While it is shown to be highly effective for miRNA classification, it is likely to be more broadly applicable. Source code is freely available on GitHub.

## Results

### Stage 1—Augmenting the labeled set using MVCT

**Fig. 1:**
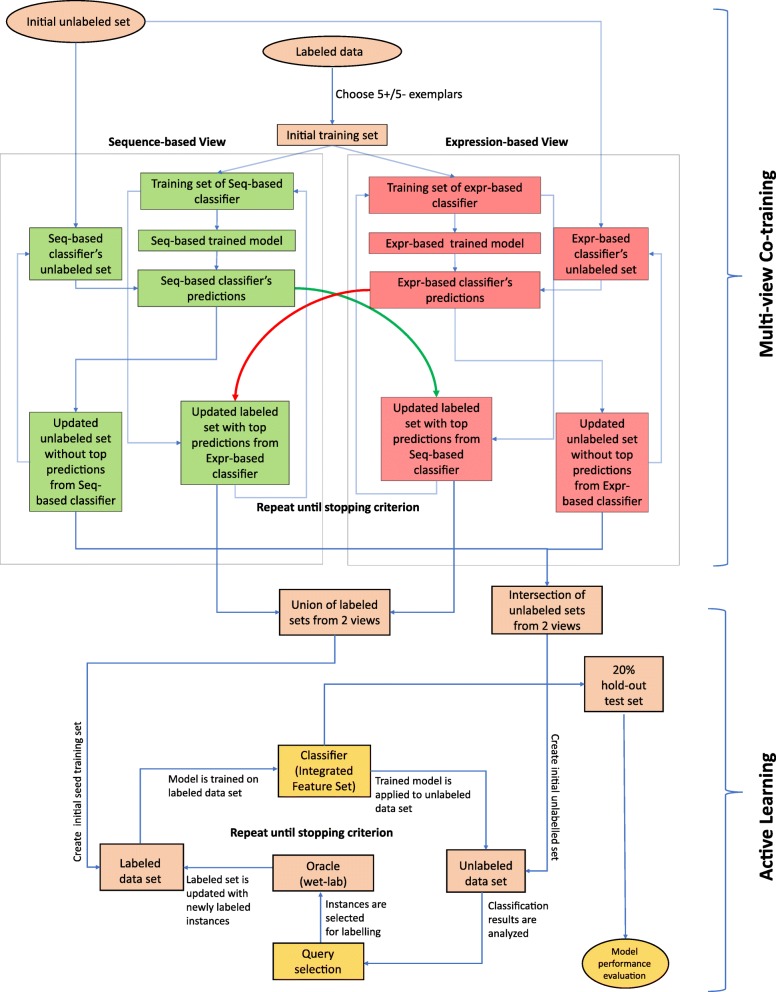
Illustration of proposed two-stage integrated semi-supervised ML classification pipeline comprising both multi-view co-training (upper) and active learning (lower)

The first stage of the integrated semi-supervised miRNA classification pipeline applies MVCT to the available training data. This is illustrated in the upper half of Fig. 1. The purpose of this step is to maximally augment the datasets representing the two views of the problem without conducting any costly wet-lab experiments. For each of the six test species (see the “Methods” section), we assess the effectiveness of applying MVCT for miRNA classification. Although the goal of stage 1 is to augment the training set for stage 2, we evaluate the performance of both the sequence and expression-based views at each iteration of learning, as an indication of the increasing value of the growing training dataset. Here, and throughout the study, performance is measured using the area under the precision-recall curve (AUPRC) over a hold-out test set (see the “Methods” section). Results are presented in Table 1, representing the mean performance of each view’s classifier averaged over 100 experiments with randomly selected seed training sets of five positive and five negative training exemplars. During each iteration of MVCT, the most confident positive and negative predictions are added to the alternate view’s training set.

**Table 1:** MVCT performance results for all six data sets over 11 iterations of learning. Results demonstrate average area under the precision-recall curves. Standard deviations were in the range of 0.001 to 0.003 for all experiments and are omitted from the table for clarity

Iteration	*hsa* exp-based	*hsa* seq-based	*mmu* exp-based	*mmu* seq-based	*dme* exp- based	*dme* seq-based	*bta* exp-based	*bta* seq-based	*gga* exp-based	*gga* seq- based	*eca* exp-based	*eca* seq-based
**–**	0.596	0.344	0.714	0.822	0.810	0.864	0.778	0.357	0.925	0.893	0.921	0.875
1	0.681	0.448	0.795	0.881	0.854	0.920	0.866	0.478	0.932	0.909	0.918	0.881
2	0.705	0.568	0.797	0.906	0.884	0.920	0.860	0.566	0.930	0.905	0.926	0.893
3	0.721	0.678	0.813	0.903	0.893	0.920	0.865	0.585	0.927	0.909	0.934	0.886
4	0.752	0.735	0.872	0.912	0.893	0.919	0.850	0.778	0.920	0.912	0.939	0.941
5	0.748	0.734	0.879	0.911	0.886	0.925	0.863	0.726	0.931	0.917	0.952	0.946
6	0.781	0.739	0.921	0.920	0.883	0.912	0.849	0.783	0.923	0.915	0.947	0.947
7	0.771	0.747	0.917	0.910	0.887	0.922	0.871	0.773	0.930	0.911	0.954	0.952
8	0.791	0.744	0.937	0.912	0.882	0.920	0.855	0.734	0.951	0.916	0.943	0.949
9	0.772	0.738	0.928	0.911	0.920	0.932	0.860	0.744	0.957	0.918	0.956	0.955
10	0.773	0.761	0.941	0.908	0.903	0.923	0.865	0.765	0.961	0.917	0.952	0.961
11	0.779	0.761	0.955	0.912	0.901	0.921	0.865	0.809	0.964	0.927	0.959	0.961

As it can be observed from Table 1, the AUPRC for both views of each species undergoes significant improvement after the 11 iterations of learning are completed. The human (hsa) and cow (bta) data sets exhibit the greatest increases in performance. For example, the human sequence-based classifier sees a 121% increase in AUPRC. MVCT appears to be least effective for the chicken (gga) and horse (eca) datasets. It should be noted that the initial classifiers for these two species were highly effective (AUPRC > 0.87) prior to application of MVCT, leaving little room for improvement. The increase in classifier performance is non-monotonic, although the trend is positive for all species. This indicates that the samples added to the training set during MVCT were correctly labeled in most instances.

**Fig. 2:**
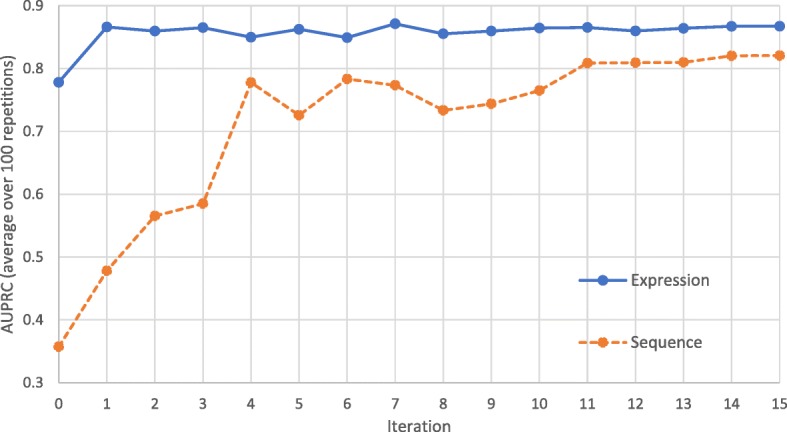
Learning curve for MVCT for *Bos taurus* (bta) showing the AUPRC for the expression- and sequence-based views over 15 iterations. Results represent the mean AUPRC observed in 100 repetitions with randomized seed training sets (5 positive and 5 negative exemplars). Performance assymptotes after 11 iterations, justifying selection of this parameter

The MVCT stage was run for 11 iterations for each species. Although dynamic stopping criteria are described in the literature, this choice was based on our previous analysis in [31] that showed that MVCT performance tends to asymptote after 11 iterations in human. This is here confirmed for cow (*bta*) using a learning curve in Fig. 2, where performance is plotted for 15 iterations of MVCT. Results represent the mean AUPRC over 100 repetitions, where a different seed training set (5+/5− exemplars) is selected for each repetition. Performance asymptotes for both views after 11 iterations, justifying this parameter choice.

### Stage 2—Active learning

In the second step, active learning was applied to the augmented labeled set resulting from MVCT in stage 1. The augmented training set is formed by the union of the training sets from each view from stage 1. By doing so, active learning was applied to an initial training set containing at most 54 labeled instances (seed set of 5+/5− exemplars, plus 11 positive and 11 negative exemplars added to each view during MVCT). Eleven iterations of uncertainty-based active learning were applied and the AUPRC results for each iteration are presented in Table 2. These results represent 100 repetitions of stage 1 and stage 2, where the starting dataset of stage 1 (MVCT) is drawn randomly in each repetition. As can be observed from Table 2, all six experiments show an increase in performance as active learning is applied, when compared to the initial round. Statistical significance (*t* test, *α* < 0.05) was established for all species except for chicken (gga), which was the second smallest dataset. The most significant performance increase is observed in the human species where, after 11 iterations of active learning, a 15.9% increase is observed in performance compared to the initial classifier. Both cow and fruit fly miRNA classifiers also substantially improved in performance, undergoing 9.6% and 2.2% increases in performance, respectively.

**Table 2:** Active learning performance results for all six data sets over 11 iterations of learning using the labeled set obtained from co-training. Results demonstrate average area under the precision-recall curves. Standard deviations were in the range of 0.001 to 0.003 for all experiments and are omitted from the table for clarity purposes

Iteration	*hsa*	*mmu*	*dme*	*bta*	*gga*	*eca*
**–**	0.779	0.955	0.921	0.865	0.964	0.961
1	0.812	0.951	0.918	0.877	0.960	0.962
2	0.856	0.959	0.921	0.890	0.963	0.965
3	0.875	0.963	0.925	0.894	0.963	0.965
4	0.881	0.963	0.928	0.916	0.964	0.968
5	0.888	0.968	0.930	0.932	0.965	0.970
6	0.891	0.970	0.929	0.939	0.965	0.971
7	0.891	0.972	0.931	0.939	0.965	0.971
8	0.896	0.972	0.937	0.941	0.964	0.971
9	0.898	0.972	0.940	0.948	0.964	0.970
10	0.901	0.972	0.941	0.947	0.965	0.971
11	0.903	0.972	0.941	0.948	0.965	0.971

**Fig. 3:**
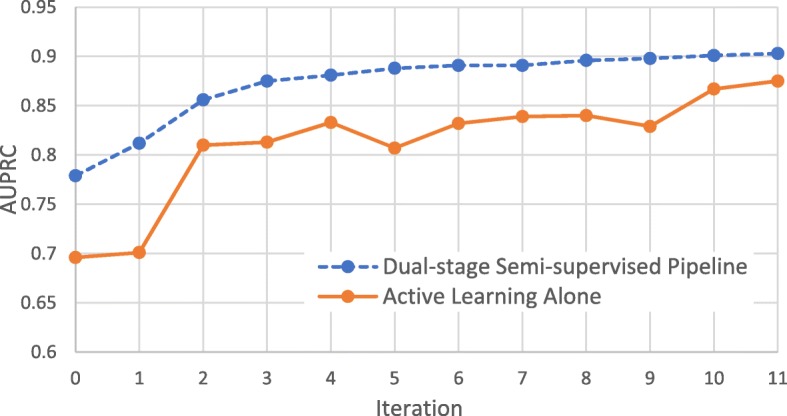
Learning curves for the human (*hsa*) dataset for active learning alone (seed training set of 5 positive and 5 negative examplars) and active learning applied to MVCT-augmented training set (i.e., proposed 2-stage integrated pipeline)

Figure 3 illustrates learning curves for two semi-supervised approaches on human: active learning alone (starting with five positive and five negative labeled training exemplars) and active learning applied to an MVCT-augmented version of the same initial training sets. As can be seen, not only does MVCT provide for an initial boost in classification accuracy, but active learning performance reaches near-optimal levels after only a small number of iterations. This illustrates the value of augmenting the starting seed training using MVCT prior to initiating the more costly active learning stage. In fact, when averaged across all six species, performance exceeding 11 iterations of active learning alone is achieved after only six iterations, effectively halving the cost of achieving the same miRNA classification performance.

**Fig. 4:**
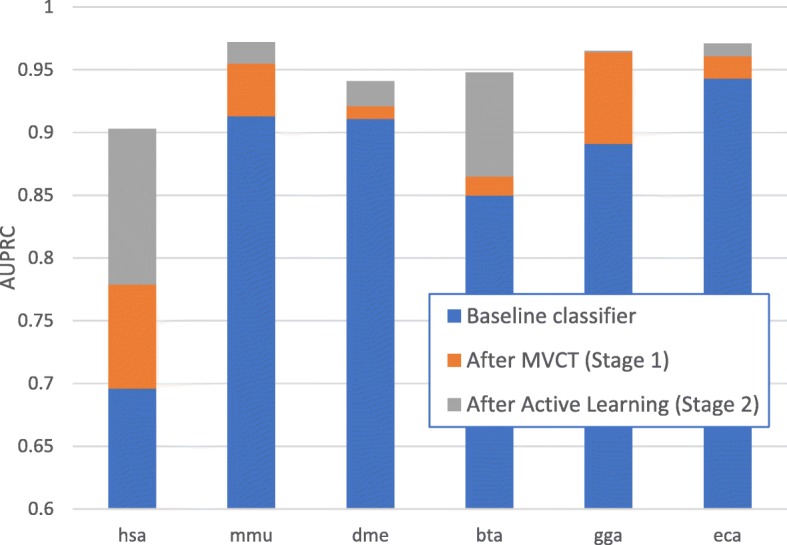
Stacked bar graphs for six test species showing relative contribution of the base classifier alone, the MVCT-augmented training set, and active learning applied after MVCT (i.e., complete pipeline)

Figure 4 illustrates the incremental value of each stage of the proposed integrated semi-supervised ML pipeline. All results in this figure represent the performance of a decision forest classifier using an integrated feature set, as used in miPIE [22]. Within each stacked bar chart, the baseline represents a classifier trained on a seed training set of only five positive and five negative exemplars. The second bar represents an equivalent classifier trained on a dataset augmented by MVCT (i.e., stage 1), while the final bar represents the same classifier after completing 11 iterations of active learning (stage 2).

From the results illustrated in Fig. 4 and reported in Table 2, it is clear that active learning has increased classification performance over all six data sets. This performance increase, however, is clearly not consistent across all data sets. The reason for this variance in performance increase can be noted in the starting performance of each data set. The initial performance reported for each data set represents the final co-training classifier after 11 iterations of MVCT. For several species, the co-trained classifier has already achieved very high AUPRC, thus leaving little room for improvement through active learning. For example, for the chicken and horse species, which are the datasets with the least increase in performance after active learning, the average AUPRC after co-training and prior to active learning is already at 0.964 and 0.961, respectively. Comparing these numbers to that of the human and cow species at 0.779 and 0.865 respectively, this inconsistency in performance increase due to active learning can be clearly attributed to the performance achieved through MVCT in stage 1. Active learning is shown to be highly complementary here; it is most effective when MVCT was least effective.

We next compared the effectiveness of our dual-stage semi-supervised method relative to two state-of-the-art miRNA classification tools: the miPIE [22] tool and the solo active learning method [32]. We here constrain these methods to 32 training exemplars in order to dedicate the same labeling budget to all methods to achieve a fair comparison. All tests on the two methods were repeated 100 times, with re-randomized data selection. Additionally, a comparison was performed against the well-known miRNA classification tool miRDeep2 [41]. miRDeep2 has previously been independently evaluated on seven data sets and shown to be one of the most effective state-of-the-art methods [42]. Since miRDeep2 has been previously trained on unrestricted training sets, we could not constrain its training data; thus, miRDeep2 represents a conservative benchmark. The results of these comparisons can be observed in Table 3. The mean and standard deviation is given for each method except miRDeep2 since it was pre-trained.

**Table 3:** Comparing average AUPRC for all six data sets over the following methods: miPIE classification tool (restricted to 32 training exemplars), miRDeep2, active learning alone, and dual stage semi-supervised pipeline. Means ± standard deviations are shown, representing 100 repetitions of each experiment (except for miRDeep2)

Data set	miPIE	miRDeep2	Active learning alone	Proposed dual-stage SS pipeline
*hsa*	0.844 (± 0.01)	0.736	0.875 (± 0.01)	0.903 (± 0.02)
*mmu*	0.966 (± 0.01)	0.915	0.972 (± 0.00)	0.972 (± 0.00)
*dme*	0.894 (± 0.01)	0.914	0.924 (± 0.01)	0.941 (± 0.01)
*bta*	0.905 (± 0.02)	0.869	0.935 (± 0.01)	0.948 (± 0.01)
*gga*	0.919 (± 0.01)	0.923	0.944 (± 0.01)	0.965 (± 0.00)
*eca*	0.919 (± 0.01)	0.843	0.971 (± 0.00)	0.971 (± 0.00)
Average	0.908	0.867	0.935	0.950

As observed from Table 3, the dual-stage semi-supervised pipeline substantially outperforms all three benchmark methods for all species tested. When compared to the miPIE and miRDeep2 miRNA classification tools, our method demonstrates an increased AUPRC of 4.2% and 8.3%, respectively, averaged over all six data sets. Also, when compared to a simple active learning approach without the benefit of a prior MVCT stage to augment its starting training dataset, our combination of co-training and active learning demonstrates an average increase of 1.5% in AUPRC. Although this increase in performance may seem relatively small, it must be considered that this is occurring at a very high-performance threshold.

## Discussion

In our experiments, we have created a dual-stage semi-supervised framework for miRNA classification with a goal of minimizing the number of costly wet-lab annotations required while maximizing classification performance. The benefit of combining both MVCT and active learning into an integrated pipeline is illustrated in Fig. 4. While we had previously examined the use of MVCT alone for increasing miRNA classification accuracy, it is used here instead to augment the training set for subsequent active learning. As illustrated in Fig. 3, the application of MVCT prior to active learning reduces by half the number of costly wet-lab annotations required to reach an equivalent performance to a traditional active learning approach. To the best of our knowledge, this study represents the first combined use of two semi-supervised ML approaches, MVCT and active learning, into an integrated classification pipeline.

Random forest (RF) classifiers are used for both MVCT and active learning classifiers in this study. This choice is based on their strong performance in previous miRNA prediction studies [18, 43]. To confirm the suitability of RF classifiers for MVCT, we did a limited comparison with a support vector machine (SVM) classifier using a linear kernel for both the expression- and sequence-based views. RF was shown to outperform SVM over each of six iterations of MVCT using human data. Therefore, RF classifiers were used throughout this study. However, we expect that these semi-supervised methods will augment the performance of any underlying classification approach.

In Table 3, our semi-supervised approach is also compared against two state-of-the-art miRNA classification tools, miPIE and miRDeep2. This comparison was performed in order to demonstrate the effectiveness of our approach in producing highly effective classifiers despite severe restrictions on the number of labeled samples required. Recall that our method does not require any costly wet-lab sample labeling experiments during the first MVCT stage, as all labels are derived from high-confidence predictions from the co-training classifiers. Thus, our entire labeling budget was limited to 32 labeled instances (10 from the initial co-training seed and 22 from the 11 iterations of active learning). Therefore, when comparing our method to the miPIE tool, we also limited its training set to only 32 labeled exemplars. Despite using the same number of labeled instances, it is observed that our method exhibits a 4.2% performance increase on average over all six data sets. When compared to the miRDeep2 tool (the de facto standard for miRNA prediction), our method outperforms this tool by 8.3% averaged over the six species. This performance increase is more impressive when one recalls that the miRDeep2 tool was trained on an unconstrained training set, whereas our method was limited to 32 labeled training exemplars, meaning we used a fraction of the tools labeled training set to obtain this improved performance. Overall, the results in Table 3 suggest that our semi-supervised pipeline approach is an effective way to train new miRNA predictors in the face of limited training data.

In the co-training step of our method, we have used a modified approach of the standard co-training model originally published by Blum et al. [24]. The original method maintains a single pool of labeled data, where the newly labeled samples from each view would be added to the single labeled pool at each iteration. In our approach, we maintain two independent labeled sets, one for each of the view, where after each iteration the newly labeled instance for one view is added directly to the other view’s labeled set. The slight variation we have applied to our co-training method reduces the risk of convergence between the two views. This is important because convergence may lead to a rapid plateau of the performance of the co-training classifier. By creating non-overlapping labeled sets for each view, we ensure that the classifiers from the two views are learning from different and independent instances

In active learning, there are two widely used strategies for query selection: certainty-based and uncertainty-based active learning. During our active learning stage, we implemented an uncertainty-based query approach, where the samples for which the classifier is most uncertain are selected for wet-lab labeling. This decision was based on findings from [32], where it was demonstrated that an uncertainty-based query approach results in higher performance for miRNA classification.

During stage 1, separate classifiers are built for each view using disjoint feature sets. However, in stage 2, a single integrated feature set is used for all classifiers, since this was demonstrated previously to be more effective than either view in isolation [22, 32]. The integrated feature set contains both sequence and expression-based features in order to leverage the predictive ability of both sets of features.

The novel combination of multi-view co-training and active learning methods proposed here offers a number of advantages. By first applying co-training to the labeled set, we are able to expand the labeled training dataset without requiring any new wet-lab annotations. Therefore, this initial growth in the training set and commensurate improvement in classification accuracy comes at no cost, beyond compute time. The only computationally expensive operations in each iteration of semi-supervised ML are the retraining of a decision forest classifier and applying that classifier to a few hundred unlabeled sequences. Neither of these operations takes more than a few minutes on a standard workstation.

Another advantage of combining MVCT and uncertainty-based active learning is that they are complementary in terms of which unlabeled data are added to the training data. Initially, the MVCT classifier adds only the high-confidence predictions from each view. Once co-training is complete, these high-confidence predictions form the seed training set for subsequent active learning, where an uncertainty-based query strategy is used, labeling only the least-confident instances. This combination of training examples allows for a wider range of evidence to be included in the training set, leading to improved classification performance.

The present study is the first reported combination of MVCT and active learning in an integrated pipeline. Other, more complex combinations of these two approaches to semi-supervised ML should be explored. For example, MVCT could be applied between each iteration of active learning to maximize the benefit derived from each round of costly wet-lab validation experiments. MVCT and active learning can also benefit from more dynamic stopping criteria based on the rate of change of the learning curve [31, 44].

## Conclusion

In this study, we have proposed a novel dual-stage semi-supervised ML approach for miRNA classification. Here, MVCT is used to augment the initial labeled training set for subsequent application of active learning. The results of this approach are shown to out-perform the state-of-the-art miRDeep2 and miPIE methods, where an increase in performance of 8.3% and 4.2% is observed in average AUPRC, respectively. A comparison is also performed against a simple active learning approach. The use of MVCT to first augment the training set is shown to be highly effective, exceeding the performance of active learning alone by 1.5%. Importantly, this increase in performance required fewer than half of the costly wet-lab validation experiments to label training data for active learning alone. Therefore, evidence gathered in this paper suggests that the proposed semi-supervised framework is a highly effective approach for reducing miRNA classification costs while increasing performance. This method will be particularly effective when studying newly sequenced genomes or non-model species where few known miRNA are available for training miRNA classifiers.

To our knowledge, this study represents the first published combination of MVCT and active learning to form an integrated dual-stage semi-supervised ML pipeline and we expect that such an approach will be effective for other applications within bioinformatics and beyond.

## Methods

### Data set selection

Five data sources were utilized for the creation of testing and training sets for each of the species in this paper: NGS expression data, genomic data, known miRNAs, known coding regions, and other known functional non-coding RNA. Expression data were drawn from small RNA NGS experiment datasets from the NCBI GEO database [45]. Genomic sequences for all known “high confidence” miRNA were downloaded from miRbase (release 22) [20]. The full genome for each of the animal species was downloaded from the UCSC genome browser database [46]. Six different species were investigated: human, mouse, fruit-fly, cow, horse, and chicken. The data are summarized in Table 4.

**Table 4:** NGS data sets examined in this study

Data set	GEO Accession	Organism	Reads	Labeled samples
*hsa*	GSM- 1820470	*H. sapiens*	38,210,937	509+/842−
*mmu*	GSM- 1528810	*M. musculus*	54,947,527	367+/844−
*dme*	GSM- 1123781	*D. melanogaster*	18,723,989	110+/97−
*bta*	GSE- 74879	*B. taurus*	43,164,654	332+/650−
*gga*	GSM- 2095817	*G. gallus*	27,937,224	193+/104−
*eca*	GSE- 100852	*E. caballus*	42,178,766	364+/224−

The true class of each sample was determined as in [32]. Briefly, miRDeep2’s “mapper.pl” preprocessing script [41] mapped each read stack from the NGS data to the reference genome of the species. This produces a set of candidate pre-miRNA complete with their sequence, secondary structure, alignment to the reference genome, and collection of NGS reads mapping to the candidate pre-miRNA. Candidates that matched known miRNA sequences from miRBase [20] annotated as true positives. All the other candidate pre-miRNA formed the candidate negative set. Candidate negative sequences were confirmed to be negative if they matched exonic sequences from known coding regions (obtained from either Ensembl [47] or the NCBI GEO database [45]). This definition of negative sequences was justified since sequences that are known to be mRNA fragments are unlikely to also form miRNA. A number of non-coding RNA (from *Rfam* [48]), that had known function other than miRNA, were added to the negative data set to ensure that the predictor would not simply learn to recognize coding regions as negatives. The *CD-HIT* [49] program was used with a 90% sequence identity threshold to remove redundant and highly similar sequences from both positive and negative datasets. Table 4 summarizes the final data set composition for each of the species. Finally, for each species, the data were split into 80% for a training set and 20% for a holdout test set.

### Feature set selection

The feature set selection approach utilized for the MVCT and active learning stages of our multi-stage approach differ slightly. Sequence-based features are obtained from HeteroMiRPred [50], including sequence-based, secondary-structure-based, base-pair-based, triplet-sequence-structure-based, and structural-robustness-related features. Eight expression-based features, derived by [22], were also included in both learners, comprising the following: (1) percentage of mature miRNA nts that are paired, (2) number of paired nts in the lower stem, (3) the percentage of RNA-seq reads in the pre-miRNA region (%reads) which are inconsistent with Dicer processing, (4) %reads that map to the loop region, (5) %reads that map to the mature miRNA region, (6) %reads that map to the miRNA* region, (7) %reads from precursor region that match Dicer processing, and (8) the total number of reads in the precursor region, normalized to experiment size.

For the co-training classifier, all eight expression-based features were used as the feature set of the expression-based classifier. To create the sequence-based feature set, the most informative sequence-based features from HeteroMiRPred [50] were selected by applying the correlation-based feature subset selection method in the Weka package [51] using default parameters to all the training data from the six animal species. Only the training portion of the data that were used for feature selection (representing 80% of the total data) from all six species were used. This algorithm seeks to minimize the correlation between selected features while maximizing their predictive ability. This results in a vector of 32 sequenced-based features pertaining to minimum free energy derived features, sequence/structure triplet features, dinucleotide sequence motifs, and structural robustness features.

For the active learning classifier, an integrated feature set was selected by applying Weka’s correlation-based feature selection to all 223 features pooled across all six species training data sets. The algorithm results in a total of 19 features including 6 expression-based and 13 sequence-based features. The selected features are MEF3, dH, Tm, Tm/loop, Sc x zG, SC/(1-dP), Probpair 1-4, 4 triplet motifs, CG, GA, #pb mature, % reads mature and % reads miRNA*. Each feature is described in detail in [22].

### Classification pipeline

All classifiers in both the active learning and co-training stages of this experiment were built using the SKLearn random forest library [52]; all parameters were set to default values except for the number of trees which was set to 500. Random forest classifiers have demonstrated excellent performance compared to other classifiers for the classification of miRNA [21, 43].

Since semi-supervised ML approaches require a small labeled dataset and a larger unlabeled dataset, we simulated this scenario in the training set by selecting a small “seed” labeled training set of five positives and five negative samples. All remaining samples were considered to be unlabeled. During the active learning stage, the oracle simply examined the known withheld label, thereby requiring no actual wet-lab validation experiments.

Our semi-supervised approach consists of two stages of learning. The first step of our approach implements a MVCT learning algorithm. Multi-view co-training makes use of multiple views of a problem to create distinct classifiers—one for each view. In the case of miRNA classification, the two views are based on the features typically used to identify miRNA: sequence-based de novo prediction or expression-based NGS prediction. Each of the sequence and expression-based classifiers is initially trained on a small seed training set of five positive and five negative labeled samples. These classifiers are then applied to the larger unlabeled data set. The sample most confidently predicted to be positive and negative from each of these views are added to the training set of the alternate view without experimental validation. Optionally, more samples could be added per iteration which may expedite convergence. We limited the MVCT algorithm to selecting only two samples in each iteration to ensure that only high-confidence predictions were being included in subsequent training sets. For each dataset, multiple learning iterations of co-training are applied in order to increase the size of the labeled set for that experiment, in order to then perform active learning on a problem with a larger labeled set. In this study, 11 iterations of MVCT are performed; dynamic stopping criteria are also available [31, 44]. Therefore, the final labeled set for each view contained 32 labeled instances.

At the conclusion of the MVCT stage, the seed training set of the active learning classifier is created by taking the union of the final labeled data sets from each view. Thus, the seed training set contains 54 labeled samples (10 from the seed, and 22 from each view of co-training). The intersection of the two MVCT unlabeled datasets forms the unlabeled dataset for subsequent active learning.

In the active learning stage, an uncertainty-based query strategy is used. Therefore, at each iteration, the single least confident positive and negative predictions will be selected for annotation by the oracle from among the unlabeled data at each iteration of learning. These will be the instances closest to the decision boundary. Once the true classes of the samples are determined, they are removed from the unlabeled set and are added to the training set. This procedure is repeated throughout the iterations. After each iteration, the model is re-trained on the new training set and the performance of the classifier is noted in the learning curve of the classifier. As with the MVCT stage, 11 iterations of active learning were completed. Performance at each iteration is computed using the 20% holdout test set. The performance of the active learning classifier represents the final performance of our method. To compute standard deviations of performance metrics, the entire dual-stage pipeline was simulated 100 times, each time starting with a different random selection for the seed training set of five positive and five negative samples. A flowchart of the described method is presented in Fig. 1.

## Data Availability

All datasets are publicly available with accession numbers listed in the manuscript. All code is available at https://github.com/GreenCUBIC/SSmiRNA.

## Abbreviations

AUPRC: Area under the precision-recall curve
bta: *Bos taurus*
dme: *Drosophila melanogaster*
eca: *Equus caballus*
gga: *Gallus gallus*
hsa: *Homo sapiens*
miRNA: MicroRNA
ML: Machine learning
mmu: *Mus musculus*
MVCT: Multi-view co-training
NGS: Next-generation sequencing
RNA: Ribonucleic acid
RT- PCR: Reverse transcription polymerase chain reaction
SVM: Support vector machine
